# Structures and Electronic Properties of Different CH_3_NH_3_PbI_3_/TiO_2_ Interface: A First-Principles Study

**DOI:** 10.1038/srep20131

**Published:** 2016-02-05

**Authors:** Wei Geng, Chuan-Jia Tong, Jiang Liu, Wenjun Zhu, Woon-Ming Lau, Li-Min Liu

**Affiliations:** 1 Beijing Computational Science Research Center, No. 10 Dongbeiwang West Road, Haidian District, Beijing 100094, China; 2 Chengdu Green Energy and Green Manufacturing Technology R&D Center, Chengdu, Sichuan, 610207, China; 3 The National Key Laboratory of Shock Wave and Detonation Physics, Institute of Fluid Physics, China Academy of Engineering Physics, P.O.Box 919-111, Mianyang, Sichuan 621900, China

## Abstract

Methylammonium lead iodide perovskite, CH_3_NH_3_PbI_3_, has attracted particular attention due to its fast increase in efficiency in dye sensitization TiO_2_ solid-state solar cells. We performed first-principles calculations to investigate several different types of CH_3_NH_3_PbI_3_/TiO_2_ interfaces. The interfacial structures between the different terminated CH_3_NH_3_PbI_3_ and phase TiO_2_ are thoroughly explored, and the calculated results suggest that the interfacial Pb atoms play important roles in the structure stability and electronic properties. A charge transfer from Pb atoms to the O atoms of TiO_2_ lead to the band edge alignment of Pb-*p* above Ti-*d* about 0.4 eV, suggesting a better carries separation. On the other hand, for TiO_2_, rutile (001) is the better candidate due to the better lattice and atoms arrangement match with CH_3_NH_3_PbI_3_.

Organic-inorganic hybrid perovskites, such as CH_3_NH_3_PbI_3_, are becoming one of the most promising materials for sunlight energy conversion because of their high efficiency, low cost, easy to prepare and solution processability^1^,^2^. Since they are firstly used as sensitizing materials in 2009 by Kojima *et al.* CH_3_NH_3_PbI_3_ has attracted increasing attentions and makes extremely fast progress in photovoltaic applications^3^,^4^,^5^,^6^,^7^. Recently, researches of perovskite-based solar cells steeply increase and the power conversion efficiency (PCE) reach rapidly to nearly 20%^8^. Several kinds of fabrication techniques were used to prepare perovskite solar cells with both mesoporous and thin-film device architectures^9^,^10^,^11^,^12^,^13^,^14^,^15^. The typical structure of perovskite-based solar cells is a layered structure with TiO_2_ layer as electron transport layer as well as CH_3_NH_3_PbI_3_ as hole transport layers. Nevertheless, the different power conversion efficiency achieved by various synthetic method implies the complexity and importance of the interface structure in efficient charge separation. For example, Yella *et al.* found that the nanocrystalline rutile TiO_2_ was much more effective in extracting photo-generated electrons from the perovskite than anatase TiO_2_ film with a higher open circuit potential^16^. Which implies the interfacial interactions are very complicated and the mechanism of interfacial electron transfer process remains unclear, thus it is important to investigate the structural, electronic properties of various perovskite interfaces.

In this study, we employed first principles method to investigate interface-related issues. Many theoretical efforts such as density functional theory (DFT) also have been devoted to study CH_3_NH_3_PbI_3_ perovskites, and partly clarified the mechanism of energy conversion in CH_3_NH_3_PbI_3_ perovskite based solar cell^17^,^18^,^19^,^20^,^21^,^22^,^23^,^24^,^25^,^26^,^27^. However, there are relatively few reports on the theoretical studies of CH_3_NH_3_PbI_3_ perovskite/TiO_2_ interface. Our previous studies investigated two kinds of CH_3_NH_3_PbI_3_ perovskite (001) and related electronic properties of the two kinds of surface^28^,^29^. Based on these, we continue our work on the CH_3_NH_3_PbI_3_/TiO_2_ interface.

## Results

The calculated cell parameters of tetragonal CH_3_NH_3_PbI_3_ perovskite are *a* = 8.94, *b* = 8.94 and *c* = 12.69 Å, which are close to the experiment values^30^. In this work, the (001) surface of CH_3_NH_3_PbI_3_ perovskite is considered, considering the CH_3_NH_3_PbI_3_ contains by the PbI_2_ and CH_3_NH_3_I (CH_3_NH_3_^+^, hereafter abbreviated as MA^+^) units, which is built along the (001). Along the *c* axis, CH_3_NH_3_PbI_3_ perovskite are composed by MAI and PbI_2_ layers, therefore, the (001) slab of perovskite owns two types of surfaces based on the different terminations: the MAI termination (MAI-T) with MA^+^ and I^−^ ions, and the PbI_2_ termination (PbI_2_-T) with Pb^2+^ and I^−^ ions. Here both terminations are considered, and seven-layer slabs is used to mimic the surface, which is thick enough to represent the surface as shown in the previous work^31^.

In the reality, the TiO_2_ is substrate to grow perovskite, therefore we use the optimized TiO_2_ cell parameters to build our supercells. Among many possible combinations between TiO_2_ and perovskite surfaces, the interfaces between rutile (001), anatase (001) and CH_3_NH_3_PbI_3_ (001) have the relatively small lattice mismatch between TiO_2_ and CH_3_NH_3_PbI_3_, thus we mainly consider the above two types of interfaces. The rutile (001) slab is represented with four layer, and the anatase (001) is consistent with five layer rotated 26.565˚ 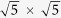 anatase surface. Coincidentally, both slabs contain 20 TiO_2_ unit, namely 120 atoms. The corresponding lattice mismatch between rutile and perovskite are −3.97%, and between anatase and perovskite are 4.56%, respectively.

**Figure 1:**
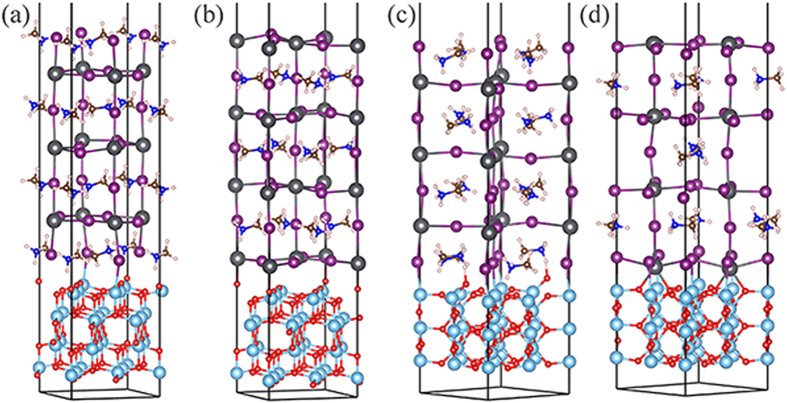
Optimized stable geometrical structures of (**a**) MAI/A, (**b**) PbI/A, (**c**) MAI/R and (**d**) PbI/R. (dark gray: lead; purple: iodine; brown: carbon; blue: nitrogen; pink: hydrogen; cyan: Ti; red: oxygen).

The TiO_2_/perovskite interfaces is built by connecting TiO_2_ slabs with seven layers MAI-T (four MAI and three PbI_2_ layers) or PbI_2_-T (four PbI_2_ and three MAI layers) slabs, as mentioned above, leaving 20 Å vacuum along the nonperiodic direction orthogonal to the surface direction. In some case, perovskite slabs were slightly shifted to avoid interaction between anion-anion, namely I-O. The system is full relaxed, and the optimized four types of interface configurations are shown in Fig. 1: Fig. 1a. MAI-T/anatase (MAI/A), Fig. 1b. PbI_2_-T/anatase (PbI/A), Fig. 1c. MAI-T/rutile (MAI/R) and Fig. 1d. PbI_2_-T/rutile (PbI/R).

As shown in Fig. 1a, the interaction between the perovskite and TiO_2_ is mainly through perovskite I atoms and under-coordinated Ti atoms of the TiO_2_ surface. The detailed bonding situation of CH_3_NH_3_PbI_3_ perovskite/TiO_2_ interface are shown in Table 1. The proportion of perovskite surface ions bonded to TiO_2_ are 50% (the bond number of one kind of atom are defined as the surface chemical bond/surface atoms number), with the bond length of 2.98 (I-Ti) and 1.73 (H-O) Å, respectively. It is also observed some structure distortion arises on the interface, for example, the shift of interlayer under-coordinated I atoms lead to the distortion of surface PbI6 octahedron. The PbI6 octahedron framework is the skeleton of CH_3_NH_3_PbI_3_ perovskite, and the bond angle between the PbI6 octahedrons (Pb-I-Pb) is rather flexible, which could decrease to 150˚ to stabilize the structure under phase change^28^; while the bond angle in the PbI6 octahedron (I-Pb-I) is rigid and always around 180˚. Here the angle of interface I-Pb-I decrease to about 174˚ relative to 176˚ in the tetragonal phase bulk, confirming the interaction of the interface. In addition, some interfacial O atoms of the TiO_2_ rise slightly with broken of original O-Ti bond to form new O-H bond. The formation of O-H bond limits the orientation and rotation of MA ions, which plays an important role in the ferroelectric domain wall of CH_3_NH_3_PbI_3_ perovskite^32^,^33^.

For PbI/A system, the formation of interfacial I-Ti bond and Pb-O bond with bond length of 3.28 and 2.33 Å. The proportion of bonded atoms for perovskite surface are 50%, which are same as MAI/A system. The interaction between them leads to a large distortion of the original surface, for example, equatorial I-Pb-I angle decrease, and partly O-Ti bond are broken. For MAI/R system, the interfacial bond types are same as MAI/A system. However, the bond lengths are slightly shorter than the MAI/A system, more importantly, all the perovskite interfacial atoms are involved in chemical bonding, indicating a better atom arrangement match between rutile (001) and perovskite than that of anatase (001). For PbI/R system, interestingly, it is observed the formation of O-Pb-O bond without the breaking of O-Ti bond. The interfacial Pb atoms obviously move towards the pervoskite, and the corresponding bond lengths of Pb-O and I-Ti are 2.4 Å and 2.9 Å, respectively. The relative shorter interfacial bond lengths indicate a strong interaction between the two surfaces. In the interfacial region, the surface PbI6 of perovskite are seriously distorted, and the opposite I-Pb-I bond angle sharply decrease to 152˚.

The calculated binding energies of the different interfaces as listed in Table 2 together with lattice mismatch and Bader charges. The binding energies of the composites were calculated by the equation:


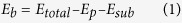


where the *E*_*total*_, *E*_*p*_, and *E*_*sub*_ denote the total energy of the perovskite/TiO_2_ system, isolated perovskite, and TiO_2_ substrate, respectively, especially, the *E*_*p*_, were calculated with allowing the cell parameter relaxation, hence the strain energy of the CH_3_NH_3_PbI_3_ slabs have been taken into account the binding energies. It is not surprising that the interfaces with PbI_2_-T slab are 0.86 and 2.82 eV more stable than the corresponding MAI-T slab for the anatase (001) and rutile (001), respectively. The reason should be that the interaction between MA ions of the MAI-T with other ions is through either van der Waals (vdW) or hydrogen bond, which is rather weak. The interaction between the Pb atom with PbI_2_-T with the other atoms is chemical bonding, which connects TiO_2_ and perovskite as a bridge.

On the other hand, the interface with rutile (001) are thermodynamically more stable than the corresponding of anatise (001) for both terminations. As shown in Table 2, the calculated binding energy of PbI/R is about 3.21 eV larger than the one of the corresponding PbI/A. The corresponding lattice mismatch between TiO_2_ and perovskite are 4.56% for anatase and −3.97% for rutile, respectively. Thus the different binding energy should come from the different lattice match between rutile and anatase TiO_2_ with perovskite (001). The lattice mismatch is relatively large between the CH_3_NH_3_PbI_3_ and TiO_2_ is relatively large, thus the strain may affect the interfacial stability between CH_3_NH_3_PbI_3_ and TiO_2_. The calculated *E*_*b*_ is negative, which suggests that the interaction between the interface atoms could compensate the mismatch energy. Mosconi *et al.* calculated the perovskite and anatase (101) interface, and they found that the lattice mismatch of pseudocubic phase perovskite with anatase (101) were small (0.75 and −1.85%) but that of tetragonal phase were large (−6.4% and −13.52%)^31^. Here we consider the tetragonal phase, which is stable under room temperature, and found the better lattice match for tetragonal phase perovskite (001) with anatase (001) as well as rutile (001).

In order to check whether the seven layer is thick enough, we also examined the binding energy with nine layers for both terminations, and the calculated *E*_b_ are within 3% compared with the one with seven layers, which indicates seven layers is thick enough to represent the properties of (001) slab of MAPbI_3_ perovskite. To summarize, the interfaces are strongly stabilized by interaction with rutile (001) than anatase (001) as well as, with interfacial Pb atoms leading to additionally higher binding energy to TiO_2_ in PbI_2_-T surface compared to that of MAI-T surface. This stability is primarily due to the presence of the oppositely charged attractive interfacing ions.

**Figure 2:**
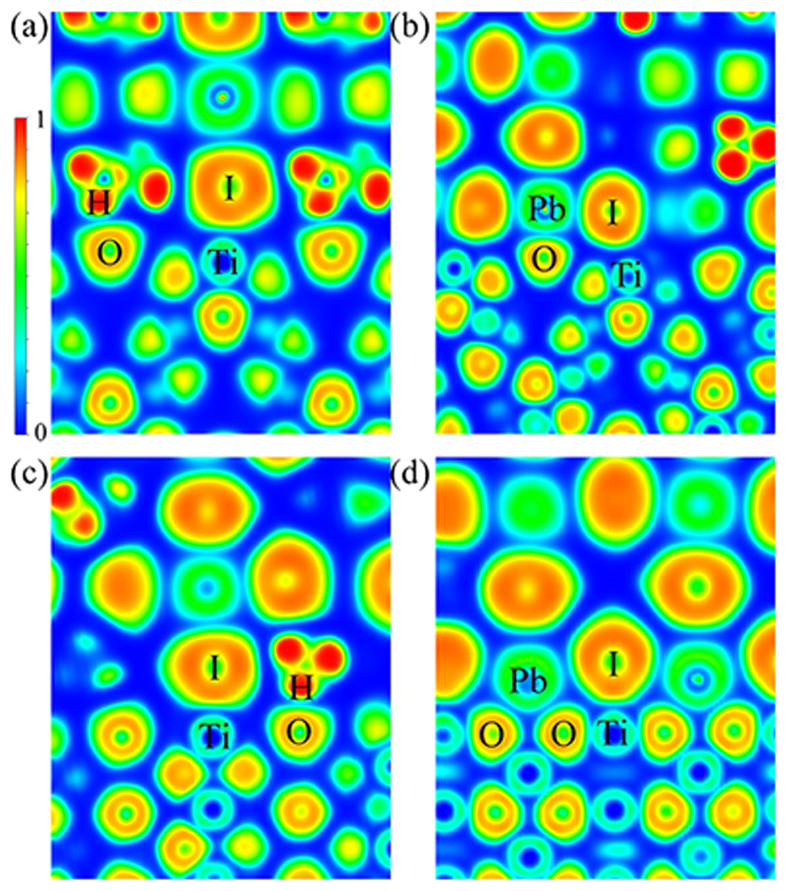
ELF o (**a**) MAI/A, (**b**) PbI/A, (**c**) MAI/R and (**d**) PbI/R.

To analyze the interactions, we have calculated the electron localization function (ELF), which can effectively reveal the nature of different chemical interactions directly from the charge localization between individual atoms. Figure 2 shows the ELF contour plots with color scheme for the four optimized CH_3_NH_3_PbI_3_ perovskite/TiO_2_ systems interfaces. The value of the ELF ranges from 0 to 1, where red color represents the electrons that are highly localized, blue color signifies electrons with almost no localization and green color with value of 0.5 corresponds to the electron-gas-like pair probability as in metallic bonds. Here, we see that in all the systems, the electros around H atoms of MA ions, I atoms and O atoms are more localized, while the electros around Pb and Ti atoms show an electron-gas-like feature. The blue color between interface anions and caions suggest the chemical interactions between them mainly origins from electrostatic interaction. Comparing Fig. 2a,c, the PbI_2_-T systems show more electron-gas-like in the interface, means a better charge transfer feature in the surface.

**Figure 3:**
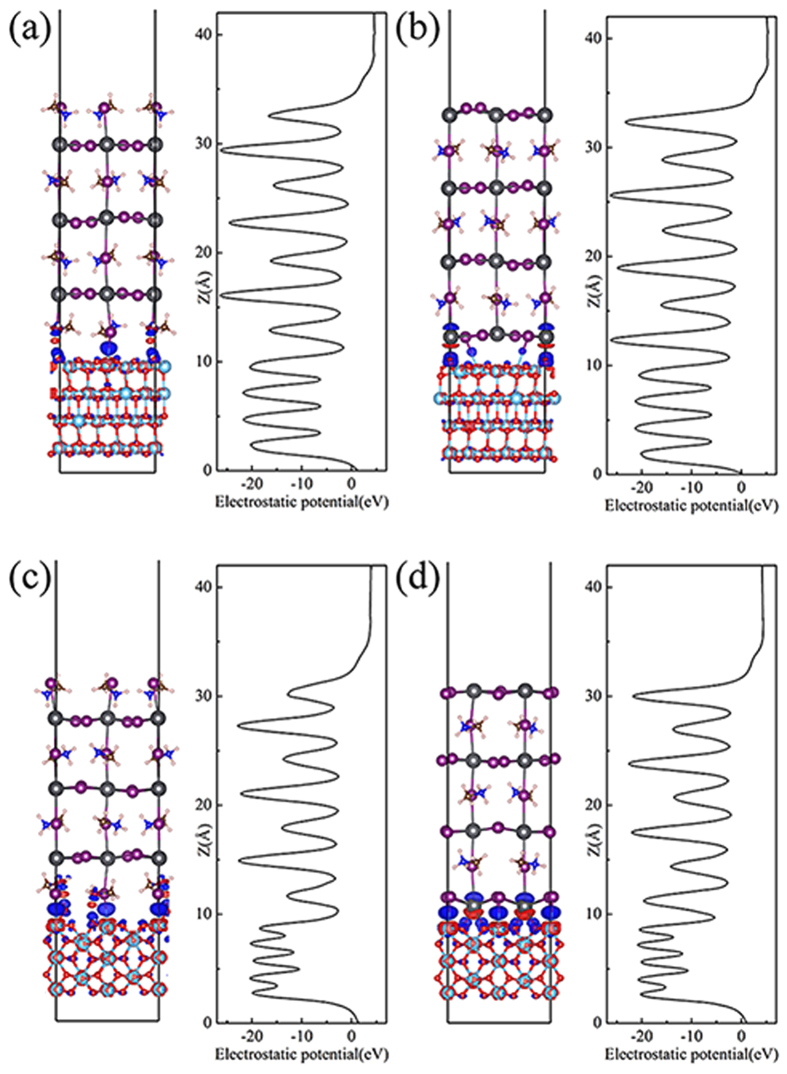
Charge density difference (left panel) and plane-averaged electrostatic potential (right panel) for (**a**) MAI/A, (**b**) PbI/A, (**c**) MAI/R and (**d**) PbI/R.

The left panel of Fig. 3 displays the difference charge density plot, i.e., the difference between the density of the perovskite/TiO_2_ system and its individual constituent, and the right panel of Fig. 3 show the plane-averaged electrostatic potential of the four structures to estimate the electronic level positions. When the TiO_2_ and perovskite forms the interface, electrons transfer from perovskite slab to TiO_2_ slab due to the Fermi level difference, therefore, it generate a built-in electric field from perovskite to TiO_2_ slab to against the charge transfer, then the interface is under equilibrium. To clarify quantitatively the charge transfer between perovskite and TiO_2_, we calculated Bader charge and which of perovskite fragment as shown in Table 2. The calculated Bader charge of perovskite fragment are −0.11, −0.07, −0.19 and −0.22 e for the four systems respectively, negative value means electron transfer from perovskite to TiO_2_. The direction of charge transfer are same for all the systems, but the charge transfer of rutile interface are larger than that of anatase.

As shown in the right panel of Fig. 3, for perovskite slabs, the electrostatic potential of MAI layers are higher than that of PbI_2_ slayers. The average potential of TiO_2_ slab is lower than that of perovskite slab, suggesting a charge transfer from perovskite to TiO_2_ in line with above results. The potential difference between rutile and perovskite slabs are relatively larger than that of anantase systems, therefore, charge transfer amount for rutile systems are larger. Since the potential of MAI layers are higher than that of PbI_2_ slayers, potential drop on the interface of PbI_2_-T systems are deeper than that of MAI-T, large amount of electrons can be accumulated to the TiO_2_ side, suggesting a better separation of electron-hole in the solar cells.

The built-in electric field behavior widely exists in the metal/semiconductor systems, the charge transfer situations of the perovskite/TiO_2_ systems are more complicated due to the variety of interface atoms arrangement. To know the details of charge transfer behavior of the perovskite/TiO_2_ systems, we draw the difference charge density plots, in which the blue color represents charge accumulation, while the red color represents charge depletion. In Fig. 3a,c, the blue color mainly distributes on the middle of I and Ti atoms, around O atoms, while the red color distributes around Ti atoms and H atoms of NH_3_. In Fig. 3b,d, the charge redistribution led by I-Ti interaction is similar as the former, and the charge redistribution around Pb atoms includes both depletion region forward to O atoms and the charge accumulation region backward to O atoms. For all the case, no redistributions is observed beyond the second layer of perovskite. Both the charge depletion and the charge accumulation make the interfacial charge redistribution, leading to the interfacial electric dipole formation. The presence of the interface dipole induce band bending, leading to the band edge alignment shift.

**Figure 4:**
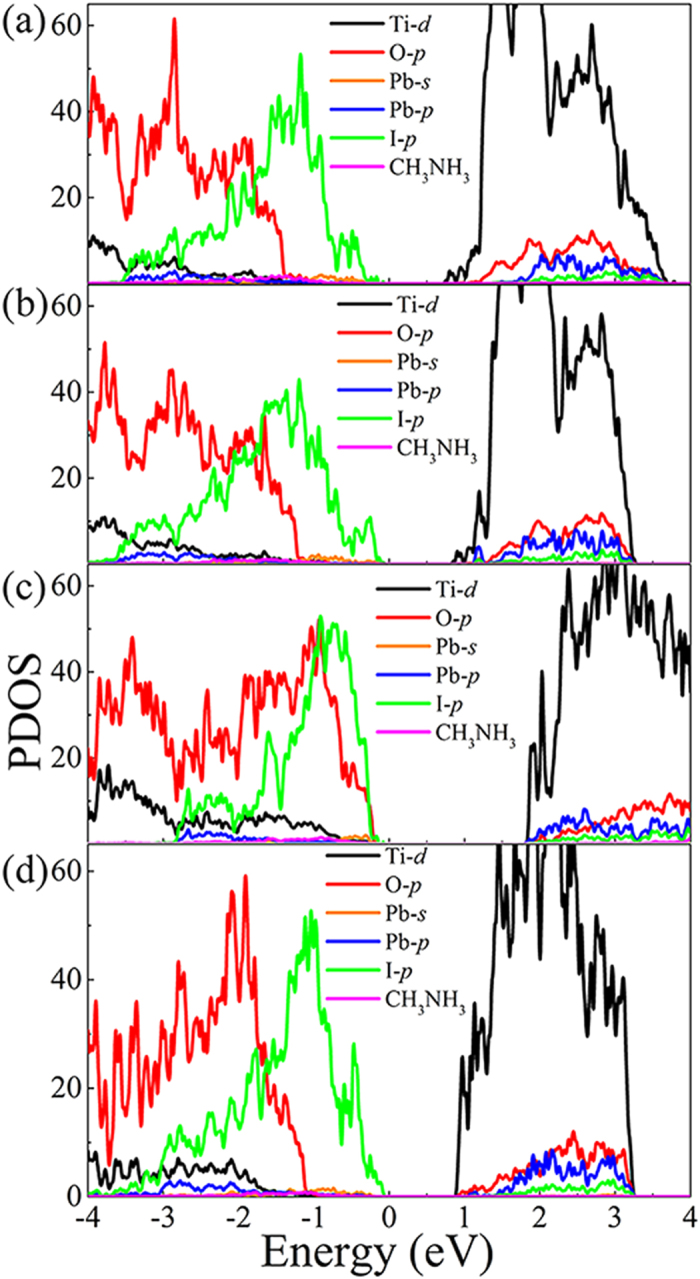
PDOS of (**a**) MAI/A, (**b**) PbI/A, (**c**) MAI/R and (**d**) PbI/R.

In order to further understand the electronic properties of the TiO_2_/perovskite interfaces, the partial density of states (PDOS) is calculated. As shown in Fig. 4, the conduction band minimum (CBM) of TiO_2_ is obviously lower than that of perovskite. Considering the bandgap of perovskite is smaller than that of TiO_2_, the electron should excite from valence band (VB) of perovskite (I-*p* and partly Pb-*s* orbital) to conduction band (CB) of perovskite (Pb-*p*), and then transfer to CB of TiO_2_ (Ti-*d*). Therefore, the energy difference between I-*p* and Pb-*p* decided the photo absorption efficiency, and the difference between Pb-*p* and Ti-*d* decided the efficiency of charge transfer cross the interface. Generally speaking, electron injection to a state lead to the left shift relative to the Fermi level, and the electron outflow are contrary. As shown in Fig. 4, the calculated band gap of PbI_2_-T is slightly smaller than that of MAI-T, because the electron outflow from the Pb atoms lead to the left shift of Pb states lower the gaps. The perovskite CB edge, contribution main from Pb-*p*, is calculated to lay about 0.7, 0.3, 0.1 and 0.2 eV above the TiO_2_ CB edge respectively for the MAI/A, PbI/A, MAI/R and PbI/R. It is noted that the band offset of MAI/A system is obviously larger than the others. This value is in line with Mosconi *et al.* calculated result of 0.8 eV obtained from a similar system of combine anatase (101) and MAI terminated perovskite (110) by GGA-DFT including SOC (spin orbit coupling)^31^. While the value of MAI/R system is slightly underestimated compared to experimental result. The PbI_2_-T systems agrees well with experimental result of 0.4 eV, which come from Lindblad *et al.* directly measuring the occupied energy levels of the MAPbI_3_ and the underneath TiO_2_^34^. It is well-known that the spin-orbit coupling (SOC) effect has great effect on the calculated band gap, as shown in the previous work^20^. In order to check the effect of the SOC, we check the SOC effect on the band gap of bulk CH_3_NH_3_PbI_3_. The calculated band gap is 0.60 eV, which is obviously smaller than the one with pure PBE and experimental results^3^. Therefore, we retain the PBE calculated of the electronic structure of the investigated interfaces, which was shown to qualitatively give the same trend as experiment.

## Discussion

In summary, we have performed first-principles calculations to study the structure and electronic properties of the interface between CH_3_NH_3_PbI_3_ and TiO_2_ including four types structures: MAI/A, PbI/A, MAI/R and PbI/R. The calculated results suggest the PbI_2_-T surface of CH_3_NH_3_PbI_3_ interact with TiO_2_ stronger due to the formation of the bridge bond. Rutile (001) surface has better lattice and atoms arrangement match with CH_3_NH_3_PbI_3_. The charge transfers from CH_3_NH_3_PbI_3_ to TiO_2_ are observed for all the four systems. The different band edge alignment show the PbI_2_-T surface and rutile (001) are better candidate for the charge separation.

## Methods

The DFT calculations were performed using the Vienna Ab Initio Simulation Package (VASP) code^35^,^36^. The electron-ion interaction was described by the projector augmented wave (PAW) method^37^,^38^,^39^. Electronic orbitals 5*d*6*s*6*p*, 5*s*5*p*, 2*s*2*p*, 2*s*2*p* and 1*s* were considered as valence orbitals for Pb, I, C, N and H atoms, respectively. The cutoff energy for basis functions was 400 eV, and the *k*-space integration was done with a 4 × 4 × 1 *k*-mesh in the Monkhorst-Pack scheme^40^. Further increasing the energy cutoff and *k*-points showed little difference on the results. All the structures considered in this study were relaxed with conjugate-gradient algorithm until the forces on the atoms were less than 0.01 eV/Å. Periodic boundary conditions were applied in all three dimensions. Due to large sizes of Pb and I ions, cages formed by four PbI_6_ octahedron are large enough to accommodate MA^+^ ions and there is no obvious chemical bond formation between MA^+^ ions and the inorganic matrix. Therefore, non-local density functional, vdW-DF^41^, was employed to take into account the weak interaction in the system, as implemented in VASP by J. Klimeš *et al.*^42^,^43^. In this method, the exchange-correlation energy takes the form of


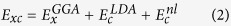


Here, the Perdew-Burke-Ernzerhof (PBE) exchange functional was employed^44^. To account for the van der Waal (vdW) interaction, the semilocal generalized gradient approximation (GGA) correlation term is replaced by the nonlocal form of “vdW correlation” 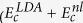 correlation energy). To improve the description of the on-site Coulomb interactions in the Ti d-states, a Hubbard correction was added (DFT+U) with an effective U parameter of 4.2 eV^45^.

## Additional Information

**How to cite this article**: Geng, W. *et al.* Structures and Electronic Properties of Different CH_3_NH_3_PbI_3_/TiO_2_ Interface: A First-Principles Study. *Sci. Rep.* **6**, 20131; doi: 10.1038/srep20131 (2016).

## Figures and Tables

**Table 1:** The proportion of bonded atoms of perovskite surface and bond length of the interface structures.

	Cation (MA^+^ or Pb^2+^)		Anion (I^−^)	
	Bond number	Bond length(Å)	Bond number	Bond length(Å)
MAI/A	50%	1.73	50%	2.98
PbI/A	50%	2.33	50%	3.28
MAI/R	100%	1.50 1.72	100%	2.88, 2.89
PbI/R	200%	2.41, 2.42, 2.38, 2.39	100%	2.88, 2.91

**Table 2:** The calculated Binding energy, lattice mismatch and Bader charge of the interface structures.

	Binding energy(eV)	Lattice mismatch	Bader charge
MAI/A	−1.01	4.56%	−0.11
PbI/A	−1.87	4.56%	−0.07
MAI/R	−2.26	−3.97%	−0.19
PbI/R	−5.08	−3.97%	−0.22
